# Notes and News

**Published:** 1888-04-07

**Authors:** 


### Notes and News.

Collected and Communicated.

The subscriptions already promised towards the proposed Derby and Derbyshire Convalescent Home amount to £2,020 5*s*. 2*d*.

The foundation-stone of the cottage hospital at Budleigh Salterton, erected as a memorial of the Jubilee, was laid by the Rev. J. Boucher.

The Earl of Strafford is to lay the foundation-stone of the cottage hospital at Barnet, on Saturday. The 3rd Middlesex Rifle Volunteers will be present, and the band of the 7th Battalion King's Royal Rifles.

It is proposed to enlarge Silloth Convalescent Institution by the addition of several small rooms, which it is thought the patients will find more home-like than large wards. The cost of the change will probably exceed £2,000, but the funds of the institution are sufficiently prosperous to justify the outlay.

In view of the discussion that has been going on with regard to the conduct of the sisters at Walsall Cottage Hospital, we think it worth while to insert *verbatim* the following letter addressed to the editor of the *Midland Evening News*: "Sir,—I feel it my duty to speak a word through your valuable paper to the public generally in respect to the Walsall Cottage Hospital, and the sisters in charge of that noble institution, having seen in your valuable paper the serious allegations that have been brought before the public from time to time. I met with an accident some time ago whilst employed by Mr. J. J. Gettings, of Goscote, which caused me to be taken to the Cottage Hospital with a leg broken in two places and badly fractured. After arriving there the broken bones were set by the sisters in charge, and I was treated the same as the rest of the patients, with the greatest love and sympathy, which characterize the sisters of that institution; and if I met with an accident to-morrow, with all due respect to surgeons and doctors, I should think mine a very bad case if those gentlemen had to be called to render their services in setting a broken bone. It has been some time now since I saw any of the sisters, but I shall always remember the attention and kindness shown towards me whilst an inmate there. If people would get the love of God shed abroad in their hearts they would feel thankful for the care and kindness shown towards them when in similar circumstances to those that I was placed in whilst an inmate of that noble institution. Hoping you will kindly insert this at your earliest convenience, I remain, sir, yours in anticipation, Jabez Brayford (late an In-patient)."

Three ladies have offered to contribute £25 a year each to maintain three cots, to be called respectively the White Dove cot, the Mentone cot, and the Amelia Lucas cot in the Bristol Sick Children's Hospital. It is expected also that the employés of a local firm will contribute £25 for the support of a cot in the hospital.

The Managing Committee of the Hull Royal Infirmary have resolved to convert the Watts Ward into a home for the nurses, who have hitherto been the least comfortably housed of any of the officials of the institution—a condition of affairs in which it is to be feared the Hull Infirmary does not stand alone.

At the annual meeting of the Hawick Cottage Hospital Dr. Hamilton stated that he thought the institution was not managed according to the intention of the founders. Chronic cases were admitted, turning the hospital into a home for incurables, and as a result of this acute cases, such as the hospital was intended for, could not be admitted for lack of space. But it is difficult always to draw a sharp line between acute and chronic cases.

The first annual meeting in connexion with the Cardiff Eye and Ear Hospital was held at the Town Hall on Thursday, the Mayor (Alderman Windsor Jacobs) presiding. In opening the proceedings, the Chairman said he regretted to find that the financial state of affairs was not so healthy as one would desire. This is a somewhat unusual occurrence in the first year of the history of a new institution. As a rule, hospitals are started with such a degree of enthusiasm as to carry them prosperously over their first decade. Do the public of Cardiff fail to appreciate the great advantage of an eye and ear hospital?

There has been a difference of opinion between the Local Board and the Guardians of Bideford, which has led to some strained feeling. At the request of the latter, pauper patients were received into the fever hospital when an outbreak of fever occurred, which threatened an epidemic; and for this the Local Board asked a contribution of £50 towards the expense of nursing and maintenance in the hospital, which amounted in all to about £200. The Guardians thought this request excessive, and offered £10, which the Board refused. The matter has been the subject of considerable discussion; but finally the Guardians have agreed to give £25 towards the hospital expenses, feeling that but for the prompt action of the Board the epidemic might have been more serious. It is certainly to be desired that important public bodies—such as Guardians—should not encourage the existing bad habit of involving hospital authorities in expense without helping them to bear the burden of it.

Provident dispensaries are attracting increased attention, both from medical men and the public. In an article in the *Provincial Medical Journal* Dr. A. W. Wallace makes several suggestions for their better management. The entrance into them is made far too easy, and many therefore enter who have no kind of justification for doing so. Individual medical men start what they call dispensaries, which, as a rule, are merely "advertised shops." These shops are conducted at a lower rate of remuneration than legitimate dispensaries, and prove the means of much injury. We do not question the right of a medical man to carry on an advertising business if it pleases him, any more than we question his right to paint himself and wear feathers like a Red Indian. We venture to remind such, however, that sometimes the longest way round is the shortest way home; and that short cuts to bread and cheese often keep a man eating very poor bread and very stale cheese for a lifetime.

THE following vacancies are announced this week, the amount of salary in each case being given after the name of the institution :—

**Resident Medical Officers.**

Liverpool Infirmary for Children, Myrtle Street. House Surgeon. —£85.
Hull Borough Asylum. Assistant Medical Officer.—£100.
Brompton Hospital for Consumption. Clinical Assistants.
Essex Lunatic Asylum. Temporary Assistant, three months.—£30.
Derby Borough Asylum. Medical Superintendent.—£350.
City of London Hospital for Diseases of the Chest. Clinical Assistant.
Central London Ophthalmic Hospital, Gray's Inn Road. House Surgeon.

**Non-resident Medical Officers.**

Westport Union. Medical Officer.—£117, and fees.
Devonport Royal Albert Hospital. Honorary Ophthalmic Surgeon.
Liddell Provident Dispensary, Jarrow-on-Tyne.—Salary £200.

**Matron.**

Derbyshire General Infirmary. One.

**Nurses.**

Bath Nurses' Home, 44, Rivers Street, Bath. Several.
Nottingham and Notts Nursing Institution. One, Medical, Surgical, and Monthly.
Birkdale Training Nurses' Home, Southport. One, Medical, Surgical, and Masseuse.
Burton-on-Trent Infirmary. One.—£23.
Kingston Nurses' Home, Baker Street, Hull. Several, Monthly, Surgical, and Massage.
Blackburn and East Lancashire Infirmary. One, Assistant.

**Probationer.**

Blackburn and East Lancashire Infirmary. One.

---

THERE has been an inquiry at Dundee Infirmary about the death of a girl shortly after her admission there, it having been suggested that death was induced by her receiving a warm bath shortly after her admission. Although it was conclusively proved that the bath in no way accelerated her decease, which was due to a wound in the lungs, the case has aroused various "ex-patients," who with one exception protest against the insult and injury they have received from these ablutions—undergoing the ordeal of the bath, as one puts it. The exception proudly boasts that he "was never washed." Apparently Scott's figurative description of his native land—"erst a slattern, and not e'en yet of cleanliness a pattern"—is still approximate; and the "ex-patients" share the feelings of the character in *The Magistrate* who, having been arrested by mistake, feels the worst of his wrongs to have been that he was "washed by the authorities."

---

THE report read to the annual meeting of the managers of the University College Hospital must be considered on the whole very satisfactory. An illuminated address was presented by the hospital to her Majesty the Queen on the occasion of the Royal Jubilee. The Queen has been patron of the hospital for half-a-century. During that time the institution has expended more than half-a-million in charity, and has afforded means of instruction to more than 6,000 medical students. The amount in legacies received during the year was £4,056, as against £2,080 in the previous year. There has been also a slight increase in the annual subscriptions, viz., from £2,048 to £2,143. The donations of the year were in excess of any previous experience, amounting to the noble sum of £10,024. Mrs. Nathaniel Montefiore contributed the handsome donation of £1,500 to the building fund. The committee recorded its deep regret at the loss of Dr. Wilson Fox, F.R.C.P., F.R.S., within the year. The debt now stands at £17,000, being slightly larger than at the commencement of the period under review. It is earnestly hoped that this formidable amount may be greatly diminished in the near future. We note with satisfaction that the cost per bed has considerably diminished since the publication of the last report, having been £59 11*s.* 5*d.* as against £62 11*s.* 5*d.* in the previous year.

THE Provident Dispensary system at Sunderland seems to be thoroughly appreciated. At its ninth annual meeting, held on Wednesday, there was a large attendance, under the presidency of the Mayor. The secretary reported that for the last four years the dispensary had had hard times to contend against, but that in spite of these the past year had been the best in its history, both financially and in point of number of entries, with one exception, since the establishment of the institution.

---

A GENTLEMAN who is interested in the Bolton Infirmary protests against the annual election of a chairman of governors of that institution, because he says "voting for a chairman from year to year would be certain ultimately to introduce politics." How or why? We do not think it possible that any sane body of men will refuse to appoint a suitable man to such a post because he happens to be a Home Ruler or an opponent of change in the House of Lords; but that may be because no medical work we have ever consulted has ever given a hint of the political tendencies indicated by scarlet fever or chronic bronchitis.

---

THE Leicester Dispensary seems to be in a rather awkward predicament. The Rev. A. A. Isaacs has brought some very serious charges against the management of the institution, and demands an inquiry, which the Governors decline to grant. They protest that Mr. Isaacs' accusations are totally unfounded—and this may be; but certainly a very serious colour is given to them by two facts—namely, that the Governors have refused a request from the members of the Dispensary, that a few of them might sit as representatives on the board of management; and that twenty-six of the medical men connected with the institution have signed a memorial stating that essential changes are necessary if the usefulness of the institution is to be maintained.

---

DR. GEORGE HARLEY is still carrying on his crusade against "moderate drinking." Everything depends on the definition of the term. What is moderate drinking? Dr. Harley makes it equivalent to constant "nipping," which falls short of drunkenness. Accepting that definition—which, however, no teetotaller would accept—we are entirely at one with Dr. Harley. Constant "nipping" is probably a very great deal more injurious to the health than a sturdy drinking bout once in two or three months. Such "nipping" we believe to be very general, and more particularly among hard worked men of business. It is one of the most serious and dangerous mistakes they can make. Most City men would be the better for total abstinence. Where that is not practicable the lightest of wines or the maturest of whiskeys in the smallest quantities not more than twice a-day should be the alternative.

---

IT seems that by a rule made sixty years ago the Vicar of Bolton is, by virtue of his office, chairman of Bolton Infirmary. Recently a proposal has been made to alter this rule, but in spite of considerable opposition it is to be retained. We consider a rule of this nature contrary to the spirit of the age and very possibly injurious to the work of the hospital. It is exceedingly rash to assume that because a man has been elected to one responsible post, he is fit for another which demands some gifts of a different kind; and so far as we can judge from the report of the annual meeting, where the suggestion was made, the present vicar himself (Canon Atkinson), whom the reformers gladly proclaim to be a most honourable, courteous, and efficient one, could have no objection to the change, which, as the Rev. S. Lees said, would probably result in his keeping the chair, not by the dead hand of an old rule, but by the warm hand of the united constituency of the meeting.

